# Timing Expression of miR203a-3p during OA Disease: Preliminary In Vitro Evidence

**DOI:** 10.3390/ijms24054316

**Published:** 2023-02-21

**Authors:** Viviana Costa, Marcello De Fine, Lavinia Raimondi, Daniele Bellavia, Aurora Cordaro, Valeria Carina, Riccardo Alessandro, Giovanni Pignatti, Milena Fini, Gianluca Giavaresi, Angela De Luca

**Affiliations:** 1 Scienze e Tecnologie Chirurgiche, IRCCS Istituto Ortopedico Rizzoli, 40136 Bologna, Italy; 2 Ortopedia Generale, IRCCS Istituto Ortopedico Rizzoli, 40136 Bologna, Italy; 3 Department of Biomedicine, Neuroscience and Advanced Diagnostics (Bi.N.D.), Section of Biology and Genetics, University of Palermo, 90133 Palermo, Italy; 4 Istituto per la Ricerca e l’Innovazione Biomedica (IRIB), 90146 Palermo, Italy; 5 Direzione Scientifica, IRCCS Istituto Ortopedico Rizzoli, 40136 Bologna, Italy

**Keywords:** osteoarthritis, microRNAs, osteoblasts, interleukines, CX-43, SP-1, TAZ

## Abstract

Osteoarthritis (OA) is a degenerative bone disease that involves the microenvironment and macroenvironment of joints. Progressive joint tissue degradation and loss of extracellular matrix elements, together with different grades of inflammation, are important hallmarks of OA disease. Therefore, the identification of specific biomarkers to distinguish the stages of disease becomes a primary necessity in clinical practice. To this aim, we investigated the role of miR203a-3p in OA progression starting from the evidence obtained by osteoblasts isolated from joint tissues of OA patients classified according to different Kellgren and Lawrence (KL) grading (KL ≤ 3 and KL > 3) and hMSCs treated with IL-1β. Through qRT-PCR analysis, it was found that osteoblasts (OBs) derived from the KL ≤ 3 group expressed high levels of miR203a-3p and low levels of ILs compared with those of OBs derived from the KL > 3 group. The stimulation with IL-1β improved the expression of miR203a-3p and the methylation of the IL-6 promoter gene, favoring an increase in relative protein expression. The gain and loss of function studies showed that the transfection with miR203a-3p inhibitor alone or in co-treatments with IL-1β was able to induce the expression of CX-43 and SP-1 and to modulate the expression of TAZ, in OBs derived from OA patients with KL ≤ 3 compared with KL > 3. These events, confirmed also by qRT-PCR analysis, Western blot, and ELISA assay performed on hMSCs stimulated with IL-1β, supported our hypothesis about the role of miR203a-3p in OA progression. The results suggested that during the early stage, miR203a-3p displayed a protective role reducing the inflammatory effects on CX-43, SP-1, and TAZ. During the OA progression the downregulation of miR203a-3p and consequently the upregulation of CX-43/SP-1 and TAZ expression improved the inflammatory response and the reorganization of the cytoskeleton. This role led to the subsequent stage of the disease, where the aberrant inflammatory and fibrotic responses determined the destruction of the joint.

## 1. Introduction

Osteoarthritis (OA) is a chronic degenerative disease characterized by progressive cartilage erosion and lesions in subchondral bone as well as in other joint tissues. The OA niche is enriched of catabolic factors, such as matrix metalloproteinases (e.g., MMP-1 and −13), aggrecans (e.g., ADAMTS-4 and ADAMTS-5), and pro-inflammatory factors/cytokines (e.g., IL-1β, IL-6, TNFα, nitric oxide, and prostaglandin E2 (PGE2)) released by synovial cells, osteoblasts, and articular chondrocytes that contribute to joint destruction and establishing aggressive inflammatory process [1,2,3,4]. The role of inflammatory processes and mediators, such as IL-1β, Toll-like receptors, IL-15/IL-17, IL-6, adipokines, collagen derivatives of nitrous oxide, and reactive oxygen species, in the initiation and progression of disease has been well studied [5]. However, the role of IL-1β might be considered controversial: (1) only a subpopulation of individuals with OA presented elevated levels of IL-1β in their synovial fluid compared with the normal individuals [6]; (2) IL-1β might have a role at the very early stages, triggering a rapid destruction of cells in the joint cartilage, but it is not clear how far its effect is extended [7]; (3) IL-1β induces apoptosis and inflammation in chondrocytes by suppression of the Nuclear factor kappaB (NF-Kb) pathway [8]; (4) IL-1β induces the upregulation of OA-relative genes and others inflammatory cytokines such as IL-6, IL-8, and tumor necrosis factor-α (TNF-α) [7,9,10], which in turn cause the release of matrix-degrading enzymes including matrix metalloproteinases (MMPs) and aggrecanases and finally lead to articular cartilage destruction [11,12]; and (5) IL-1β induces the upregulation of miRNA [13] and consequently regulation of cell functions.

Based on the evidence of these effects, it could be hypothesized that the regulation of IL-1β signaling was activated by the cooperation of different proteins and/or miRNAs. It is known that the regulation of the inflammatory process in the OA niche is mediated by the involvement of cell-to-cell communication through gap junction proteins or through the release of exosomes enriched by miRNAs or proteins; one of these proteins is Connexin 43 (Cx-43) [14,15,16,17,18,19]. Cx-43 displays many cell functions, including cell proliferation, migration, and differentiation, and it is involved in wound healing and inflammation. Regarding OA disease, it is demonstrated that Cx-43 is involved in the (1) nuclear translocation of Twist-1, improving the chondrocyAte-mesenchymal transition; (2) increase in the expression of proinflammatory mediators; (3) interaction with the astroglial– mesenchymal transition via nuclear translocation of the Yes-associated protein (YAP), a potent transcription coactivator of the cell differentiation process; (4) alteration of the recruitment of specificity protein 1 (Sp-1) into specific promoter binding sites of TWIST and COL2A-1 genes; and (5) Cx-43 involvement in the regulation of Sp-1 recruitment in OA osteoblasts and chondrocyte-derived cells by the regulation of miR-31-5p and miR-33a-5p [20]. In addition, Varela-Eirín et al. [21] revealed that the small extracellular vesicles (sEVs) released by human OA-derived chondrocytes contained high levels of Cx-43 and induced a senescent phenotype in the targeted chondrocytes and synovial and bone cells contributing to the formation of an inflammatory and degenerative joint environment by the secretion of senescence-associated secretory associated phenotype (SASP) molecules, including IL-1β, IL-6, and MMPs.

Progressive joint tissue degradation and loss of extracellular matrix (ECM) elements, along with varying degrees of inflammation, are important hallmarks of OA disease. The identification of specific biomarkers to measure the various stages of the disease becomes a primary necessity in clinical practice. This need combined with the possibility of using multiple sources for the identification of biomarkers, such as urine, serum, biopsy tissue, and synovial fluid, led to an acceleration of these studies. For example, the possibility to detect microRNAs in tissues, cells, or blood with different methods makes them an excellent method for identifying key biomarkers for the step-by-step diagnosis and understanding of the disease [6,22,23].

MiRNAs are small non-coding RNAs that are part of the miRNA-induced silencing complex (RISC) and are involved in the regulation or deregulation of the gene expression of numerous physiological processes and pathological conditions [20,24,25,26]. A recent meta-analysis study reported the major role of 27 miRNAs and their targets in OA progression [22]. MiR-140 and miR-199 are two downregulated miRNAs in the synovial tissues of OA patients compared with healthy controls that were identified. Their expressions have been shown to decrease during OA and have been inversely correlated with the severity of disease [12,27]. MiR-22 that targets BMP7, a factor inducing chondrocyte terminal differentiation, and miR-27b that targets MMP13, a key remodeling enzyme in hypertrophic terminally differentiated chondrocyte [28], were also identified as mediators of the middle stage of OA progression. Recently, our results also contributed to the understanding of the role of different miRNAs in the differentiation of human mesenchymal stromal cells (hMSCs) into osteoblasts and in the OA disease. We identified the miR-675-5p, miR-31-5p, and miR33a family as modulators of hMSC osteoblast differentiation, LIPUS-mechanosensitive miRNA, and regulators of YAP and EGFR signaling in the differentiation of hMSCs into osteoblasts, respectively. In addition, miR-33a-3p and miR-33a-5p were identified as mediators of the different expression of CX-43 and SP-1 in osteoblasts and chondrocytes derived from patients with OA [20,24,25,26].

The present study aimed to highlight the role of miR203a-3p during the evolution/progression of OA as a possible biomarker of disease, through the identification of the related molecular mechanisms in which miR203a-3p was involved during the progression of OA. Here we performed our investigations starting from evidence recovery from osteoblasts in OA patient-derived cells to hMSCs treated with IL-1β to mimic different OA progression stage in vitro [9]. MiR203a-3p was well identified as possible tumor suppressor miRNA because it is able (1) to improve apoptosis signaling through the downregulation of ZNF217 in colorectal cancer [29]; (2) to regulate ERα signaling in endometrial carcinoma in which blocked or modified cell proliferation [30]; and (3) to alter the expression of Smad9 in MSCs derived from multiple myeloma patients, which modified the osteogenic differentiation ability [31]. However, only a few studies have investigated the biological effects of miR203a-3p in bone disease until now [31,32,33,34]. While miR203a-3p regulates the transition from osteogenic to adipogenic differentiation of hMSCs in postmenopausal osteoporotic microenvironments, downregulating its target gene DKK1, it appears to have different actions in OA [35]. It was demonstrated that (1) it enhances cellular inflammatory responses and cell damage and reduces aggrecan and Col2A1 levels [9]; (2) it binds with ERα and exerts its effects in OA development through this axis [12]; (3) it is promoted by IL-1β stimulation leading to chondrocyte injury, improving the inflammation process and diminishing aggrecan and Col2A1 expression [36]; and (4) miR203a expression is dysregulated in the knee articular cartilage of OA patients compared with the controls in three or more independent studies [12]. Overall, in this study, we investigated the role of miR203a-3p and the related molecular mechanisms in which it was involved during OA progression, such as the inflammatory response and the reorganization of the cytoskeleton, starting from evidence obtained by osteoblasts derived from OA patients to hMSCs treated with IL-1β to mimic in vitro different OA progression stages [6].

## 2. Results

### 2.1. Inflammatory Conditions of OA Patient-Derived Cells

To understand the inflammatory conditions of OBs isolated from OA patients with different levels of KL grades (OB-KL ≤ 3 and OB-KL > 3) [37], we evaluated the expression of IL-1β, a master pro-inflammatory cytokine, IL-6, and IL-8 by qRT-PCR analysis. The gene expression analysis of these soluble factors revealed that OBs expressed them at different levels based on the grade of OA disease. In fact, OB-KL > 3 showed higher levels of *IL-1β* and *IL-8* compared with OB-KL ≤ 3; on the contrary, *IL-6* was upregulated in OB-KL ≤ 3 compared with OB-KL > 3. However, the released IL-6 and IL-8 proteins both were increased in the OB-KL > 3 group compared with OB-KL ≤ 3. These data were opposed to those of mRNA expression, probably suggesting the different mRNA methylation patterns on their promoter genes [38,39].

### 2.2. miRNAs Induced by Inflammatory Microenvironments on OB-OA-Derived Cells

**Figure 1:**
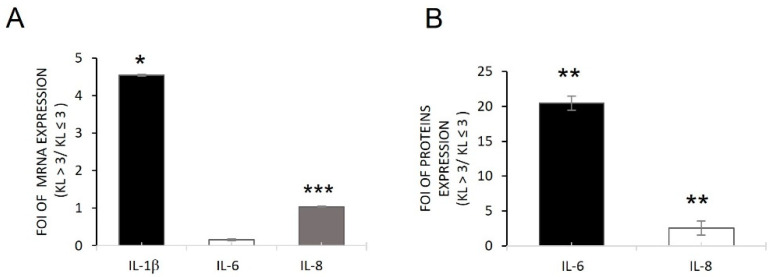
Interleukin expression in osteoblasts derived from OA patients. qRT-PCR (**A**) and ELISA (**B**) analysis of IL-1β, IL-6, and IL-8 gene expression in OBs derived from OA patients. Data are reported as FOI of expression between the KL > 3 group/KL ≤ 3 group (mean ± SD, n = 4). Student’s *t*-test: *, *p* < 0.05; **, *p* < 0.005; ***, *p* < 0.0005.

**Figure 2:**
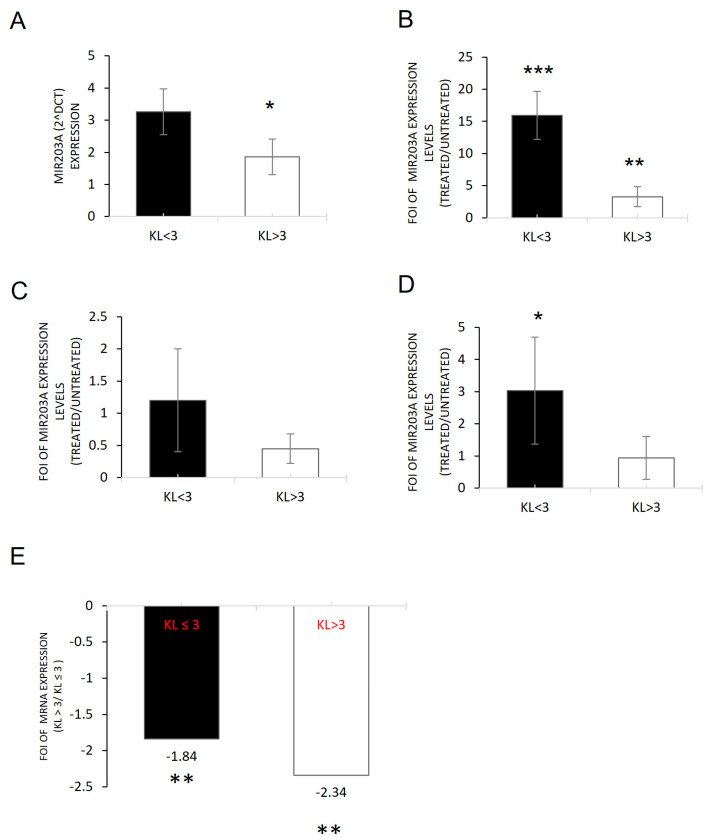
miR203a-3p expression and modulation in osteoblasts derived from OA patients. qRT-PCR analysis of miR203a-3p basal expression on OBs derived from the KL > 3 group and the KL ≤ 3 group. Data are reported as 2^−Δt^ (mean ± SD, n = 4). Student’s *t*-test *, *t* = 3.12, *p* = 0.021 (**A**). miR230a-3p expression analysis on (**B**) OB-derived cells after treatment with IL-1β, (**C**) after transfection with miR203a-3p inhibitor, and (**D**) after co-treatments IL-1β and miR203a-3p inhibitor. Data are reported as FOI of expression between treated and untreated samples. (**E**) qRT-PCR analysis of miR203a-3p expression in OBs co-treated with IL-1β and miR203a-3p inhibitor. Data are reported as FOI of expression between co-treated cells compared with IL-1β-treated cells (mean ± SD, n = 4). One-way ANOVA (KL ≤ 3: *F* = 48.2 *p* < 0.0005; KL > 3: *F* = 7.30, *p* = 0.002); multiple comparisons versus untreated (* *p* < 0.05; ** *p* < 0.005; *** *p* < 0.0005).

It is reported that IL-1β regulates the miR203a-3p expression during different diseases [3,10,12,40,41,42]. To investigate this evidence and the data reported in Figure 1, we performed qRT-PCR analysis on OB cells derived from OA patients (OB-OA). As shown in Figure 2, both cells expressed miR203a-3p, which was significantly higher in the OB-KL ≤ 3 group compared with the OB-KL > 3 group. To investigate the involvement of IL-1β on miR203a-3p expression, we treated OA-derived cells with IL-1β at 20 ng/mL for 48 h (Figure S1) [12]. The qRT-PCR analysis demonstrated the ability of these cells to express high levels of miR203a-3p after IL-1β treatments, compared with untreated groups (Figure 2B). Through a gain and loss of function study on OBs derived from patients, these data were verified. We overexpressed miR203a-3p inhibitor in OBs, and as expected, miR203a-3p was downregulated after transfection compared with untreated cells (Figure 2C); this was also true after co-treatments of OBs derived from OA patients with IL-1β and miR203a-3p inhibitor transfection. As shown in Figure 2D, miR203a-3p was downregulated after co-treatment compared with untreated groups and compared with IL-1β-treated cells (Figure 2E).

**Figure 3:**
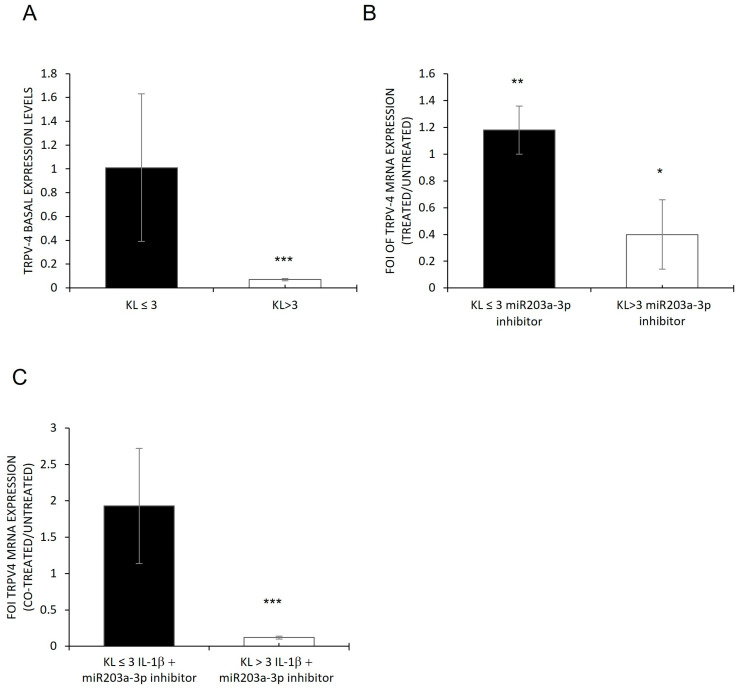
Evaluation of TRPV4 expression on osteoblasts derived from OA patients. qRT-PCR analysis of *TRPV4* mRNA expression on OBs derived from the KL > 3 group and the KL ≤ 3 group (**A**) and transfected with miR203a-3p inhibitor (**B**) and co-treated cells versus untreated (**C**). Data are reported as FOI of expression (mean ± SD, n = 4). One-way ANOVA (KL ≤ 3: *F* = 6.74, *p* = 0.003; KL > 3: *F* = 24.37, *p* < 0.0005); multiple comparisons (* *p* < 0.05; ** *p* < 0.005; *** *p* < 0.0005).

To understand the role of the current miRNA data, a bioinformatic investigation through a target prediction scan was performed, revealing that miR203a-3p targets different genes involved in the OA process or OB differentiation. To validate these bioinformatic data, the expression levels of TRPV4 were evaluated on OB-OA-derived cells [43,44,45]. Figure 3 shows that OB cells had a low level of this miRNA target gene, and its expression was restored after miR203a-3p inhibitor transfection (A) or after co-treatments with IL-1β plus miR203a-3p inhibitor transfection (B) compared with untreated groups.

**Figure 4:**
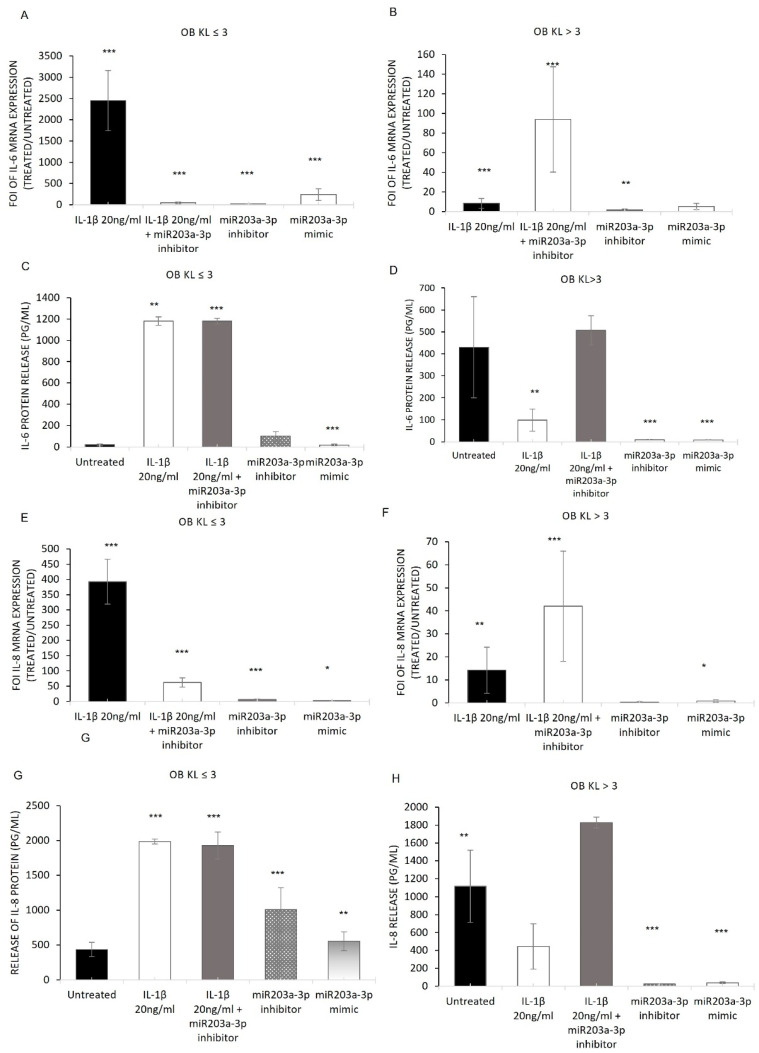
Gain and loss of function studies on osteoblasts derived from OA patients. Results of qRT-PCR (**A**,**B**,**E**,**F**) and ELISA (**C**,**D**,**G**,**H**) analysis of *IL-6* and *IL-8* mRNA expression levels on OBs derived from patients with KL ≤ 3 and KL > 3 treated with IL-1β and co-treated with IL-1β and inhibitor transfection, miR203a-3p inhibitor transfection, and miR203a-3p mimic transfection (mean ± SD, n = 4). One-way ANOVA; multiple comparisons versus untreated (* *p* < 0.05; ** *p* < 0.005; *** *p* < 0.0005).

To understand the link between miR203a-3p expression and inflammatory interleukins in OA disease, we first investigated the modulation in terms of mRNAs and the protein releases of IL-6 and IL-8 in OB-OA samples through gain and loss of function studies. The data obtained from IL-6 gene and protein expression (Figure 4A–D) showed that IL-1β alone and in co-treatments with miR203a-3p was able to induce the upregulation of IL-6 mRNA and protein compared with untreated groups; in particular, the co-treatments with IL-1β and miR203a-3p inhibitor improved a strong upregulation of the IL-6 protein release. In contrast, mimic and inhibitor transfection induced a lower upregulation compared with untreated cells of IL-6 mRNA and proteins in both OB groups.

The analysis of the IL-8 expression after treatments with IL-1β, transfection with miR203a inhibitor or mimic, and co-treatments of IL-1β and miR203a-3p inhibitor showed the same modulation revealed for the IL-6 mRNA and protein released in both OB-OA-derived cells (Figure 4E–H).

### 2.3. hMSC Evaluation of IL-1β Stimulation: In Vitro Model of OA Disease

**Figure 5:**
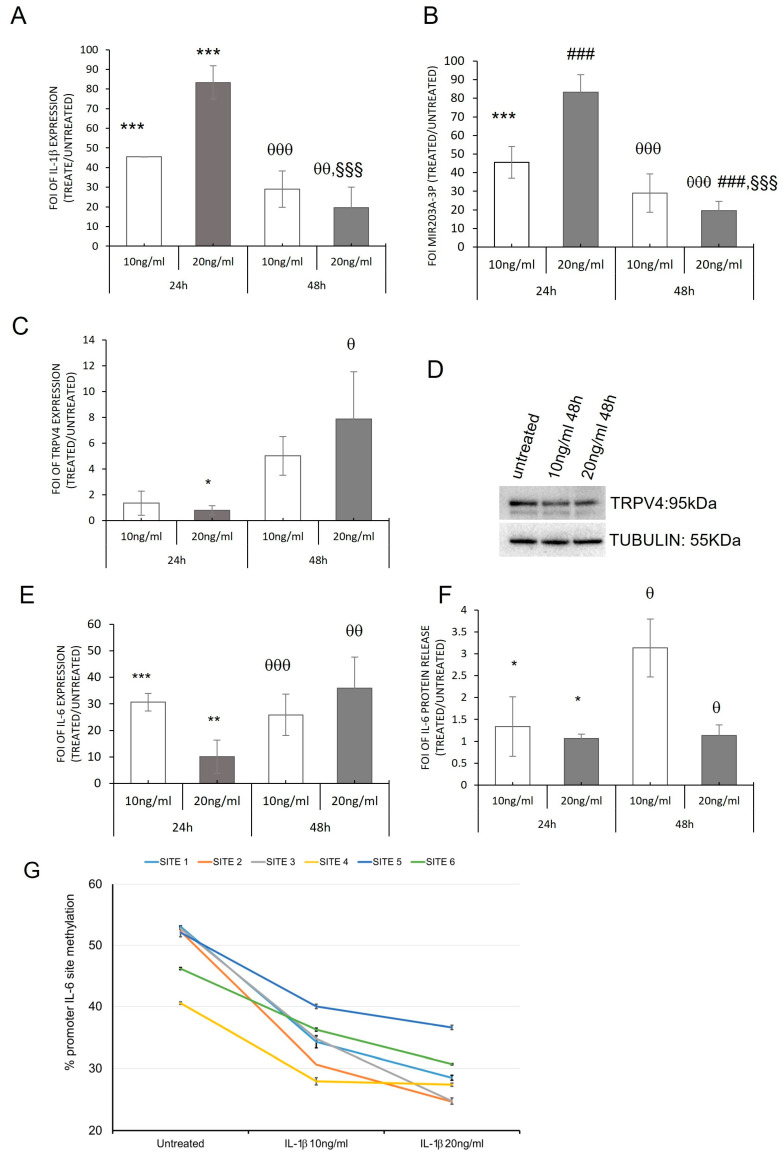
Effects of IL-1β treatments on hMSCs. qRT-PCR analysis of IL-1β mRNA (**A**), miR203a-3p (**B**), TRPV4 (**C**), and IL6 (**E**) expression on hMSCs treated with IL-1β (10 ng/mL and 20 ng/mL) for 24 h and 48 h. Data are expressed as fold of induction (FOI) in gene expression (2^−ΔΔCt^) occurring in treated vs. scrambled untreated groups (mean ± SD, n = 4). Western blot (**D**) of TRPV4 on hMSCs treated with IL-1β (10 ng/mL and 20 ng/mL) for 48 h and ELISA assay for IL6 quantification (**F**) obtained by the same samples. Two-way ANOVA; multiple comparisons (one symbol, *p* < 0.05; two symbols, *p* < 0.005; three symbols, *p* < 0.0005) versus: *, untreated 24 h; ^θ^ untreated 48 h; ^#^ IL-1β 10 ng/mL 24 h; ^§^ IL-1β 20 ng/mL 24 h. (**G**) Percentage of methylation of six methyl-sensible restriction sites through MSRE-PCR analyses of IL-6 promoter of hMSCs after 48 h under the following treatments: untreated, IL-1β (10 ng/mL), and IL-1β (20 ng/mL) (mean ± SD, n = 3, duplicates, sites 3, 5, and 6: *p* < 0.0005; sites 1 and 2, *p* < 0.05; site 3, NS).

To validate the correlation between inflammation and miR203a-3p expression in OA disease, we investigated this relationship on the hMSC model of OA. We performed an in vitro evaluation on hMSCs treated with IL-1β at two different doses for 24 h and 48 h [33]. qRT-PCR analysis revealed that hMSCs presented an increase in IL-1β over time, in particular at 24 h, compared with the related untreated groups (Figure 5A), while showing the same increase in both doses of treatments after 48 h. Regarding miR203a-3p expression, it increased over time and was particularly higher in hMSCs treated with IL-1β 20 ng/mL for 24 h compared with the 48 h treatments, in which hMSCs maintained the upregulation of it but in a different amount (Figure 5B). The functionality of miRNA was demonstrated by the downregulation of TRPV4, in terms of RNA (Figure 5C) and protein (Figure 5D).

To understand the role of IL-1β as a modulator of IL-6 release, a gene expression analysis of *IL-6* was carried out on hMSCs treated with IL-1β to highlight the differences between the experimental times. As shown in Figure 5E, hMSCs displayed the similar trend of IL-6 expression that was displayed for the OBs derived from OA patients (Figure 1A,B); in fact, a low increase in mRNA and a strong upregulation of interleukin released compared with the untreated group (Figure 5F) were revealed at the same experimental times (*p* < 0.05).

To better understand these differences, we performed a methylation analysis of the *IL-6* promoter gene. The obtained data (Figure 5G) showed a downregulation after 48 h of treatments of its promoter methylation, justifying the different expression of IL-6 mRNA and protein revealed after treatments.

### 2.4. Involvement of IL1-β and miR203a-3p in CX-43 Expression

**Figure 6:**
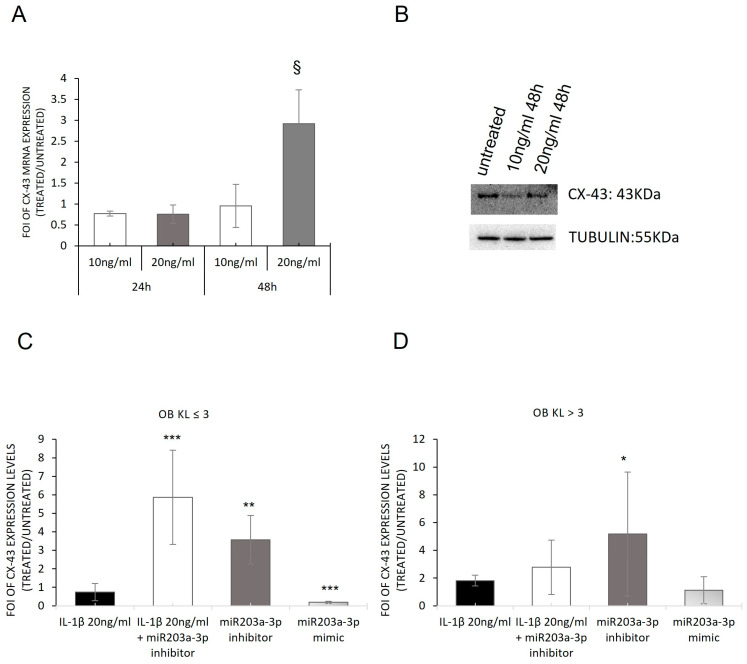
Involvement of IL-1β in CX-43 expression on treated hMSCs and osteoblasts derived from OA patients. qRT-PCR analysis of *CX-43* mRNA expression on hMSCs treated with IL-1β (10 ng/mL and 20 ng/mL) for 24 h and 48 h (**A**) and OB-OA-derived KL ≤ 3 (**C**) and KL > 3 (**D**) groups treated with IL-1β, miR203a-3p inhibitor, and miR203a-3p mimic or after co-treatments with IL-1β and miR203a-3p inhibitor. Data are expressed as fold of induction (FOI) in gene expression (2^−ΔΔCt^) occurring in treated vs. scrambled untreated groups (mean ± SD, n = 4). Two-way (treatment and experimental time interaction for hMSC: *F* = 3.70, *p* = 0.045) and one-way ANOVA (KL ≤ 3: *F* = 42.75 *p* < 0.0005; KL > 3: *F* = 2.17, *NS*); multiple comparisons (one symbol, *p* < 0.05; two symbols, *p* < 0.005; three symbols, *p* < 0.0005) versus: *, OBs; ^§^, hMSCs untreated 48h. Western blot of CX-43 and tubulin expression on hMSCs treated with IL-1β (10 ng/mL and 20 ng/mL) for 48 h (**B**).

Starting from the evidence about the role of Cx-43 in bone regeneration processes and as a modulator of inflammatory signaling through the activation of the NF-κB cascade [18,46], we investigated its involvement in hMSCs treated with IL-1β and in OB-OA-derived cells. The data reported in Figure 6 showed the different expressions of CX-43 levels during the experimental time points. After 48 h of treatments with IL-1β at the concentration of 20 ng/mL, hMSCs expressed high levels of CX-43 in terms of mRNA and protein compared with untreated cells (Figure 6A,B), while after 24 h of treatments, no modulation of CX-43 was observed compared with the untreated group. Through a gain and loss of function study on OBs, these data were verified. We overexpressed miR203a-3p in OBs, and as expected, a downregulation of *CX-43* mRNAs levels (Figure 6C,D) in OBs transfected with miR203a-3p mimic was found compared with untransfected cells, following the same modulation highlighted after treatments with IL-1β. On the contrary, the overexpression of miR203a-3p inhibitor induced an increase in *CX-43* expression into both OB-OA groups compared with those untransfected. These are also confirmed through the overexpression of miR203a-3p inhibitor after IL-1β treatment, in which OB-OA patient- derived cells, in OBs derived from patients with KL ≤ 3, improved the expression of *CX-43* mRNA levels compared with untreated or IL-1β-treated cells (Figure S2).

### 2.5. Modulation of SP-1 Expression in OB-Derived OA Patients

**Figure 7:**
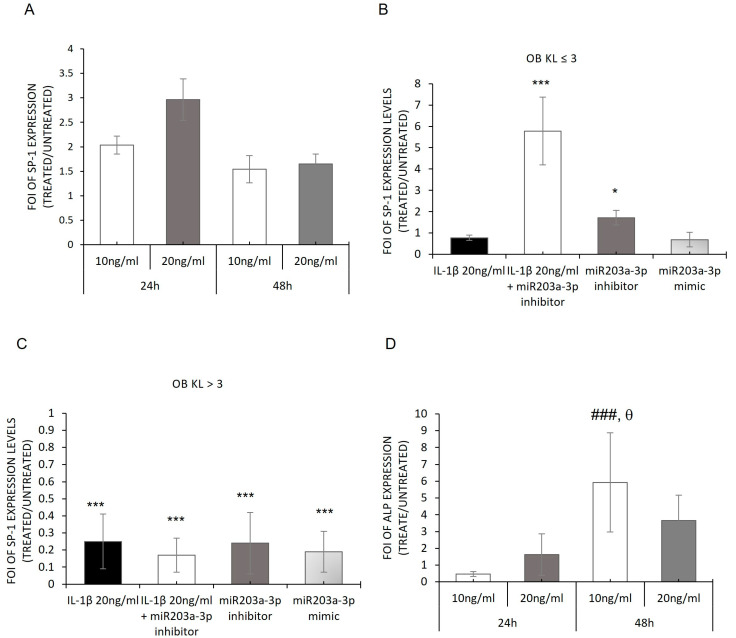
SP-1 expression: from hMSCs treated with IL-1β to gain and loss of function study on OBs derived from OA patients. qRT-PCR analysis of *SP-1* mRNA expression on hMSCs treated with IL-1β (10 ng/mL and 20 ng/mL) for 24 h and 48 h (**A**) and on OB-OA-derived KL ≤ 3 (**B**) and KL > 3 (**C**) groups treated with IL-1β miR203a-3p inhibitor, and miR203a-3p mimic and after co-treatments IL-1β and miR203a-3p inhibitor. qRT-PCR analysis of *ALP* expression on hMSCs treated with IL-1β (10 ng/mL and 20 ng/mL) for 24 h and 48 h (**D**). Data are expressed as fold of induction (FOI) in gene expression (2^−ΔΔCt^) occurring in treated vs. scrambled untreated groups (mean ± SD, n = 4). Two-way (SP1: treatment effect, *F* = 31.82, *p* < 0.0005; experimental time effect, *F* = 10.64, *p* = 0.004; ALP: treatment and experimental time interaction, *F* = 6.52, *p* = 0.007) and one-way ANOVA (*SP1*, KL ≤ 3: *F* = 39.58, *p* < 0.0005; KL > 3: *F* = 30.23, *p* < 0.0005); multiple comparisons (one symbol, *p* < 0.05; two symbols, *p* < 0.005; three symbols, *p* < 0.0005) versus: *, OB; ^θ^, untreated 48 h; ^#^ IL-1β 10 ng/mL 24 h.

It is reported that Sp-1 regulates the CX-43 gene promoter in physiological and pathological conditions [20], and its expression is dependent on the amount of CX-43 at the cell membrane [15,47,48]. To investigate the regulative role of IL-1β on the *SP-1* gene, its expression was evaluated in hMSCs treated with IL-1β and in OB-OA cells. As shown in Figure 7A, *SP-1* mRNA was upregulated in hMSCs at each experimental time point compared with the untreated group. Through the gain and loss of function studies, we highlighted that *SP-1* mRNA was significantly upregulated in the OB-KL ≤ 3 group (Figure 7B) after transfection with miR203a-3p inhibitor but also in a significant manner after co-treatments (IL-1β plus miR203a-3p inhibitor). While in the OB-KL > 3 group, SP-1 was downregulated independently by the treatment (Figure 7C), suggesting the probable role of *SP-1* as a mediator of miR203a-3p signaling during the early stage of the OA disease in which the amount of miR203a-3p was higher compared with the severe stage of OA.

Following these data, we evaluated the expression of one gene target of SP-1, Alkaline phosphatase (*ALP*), a specific osteoblast marker and an SP-1 target gene [49], on hMSCs after IL-1β stimulation; an upregulation of *ALP* mRNA compared with untreated cells (*p* < 0.0005) was identified (Figure 7D).

### 2.6. Modulation of YAP and TAZ Complex by IL-1β Stimulation

**Figure 8:**
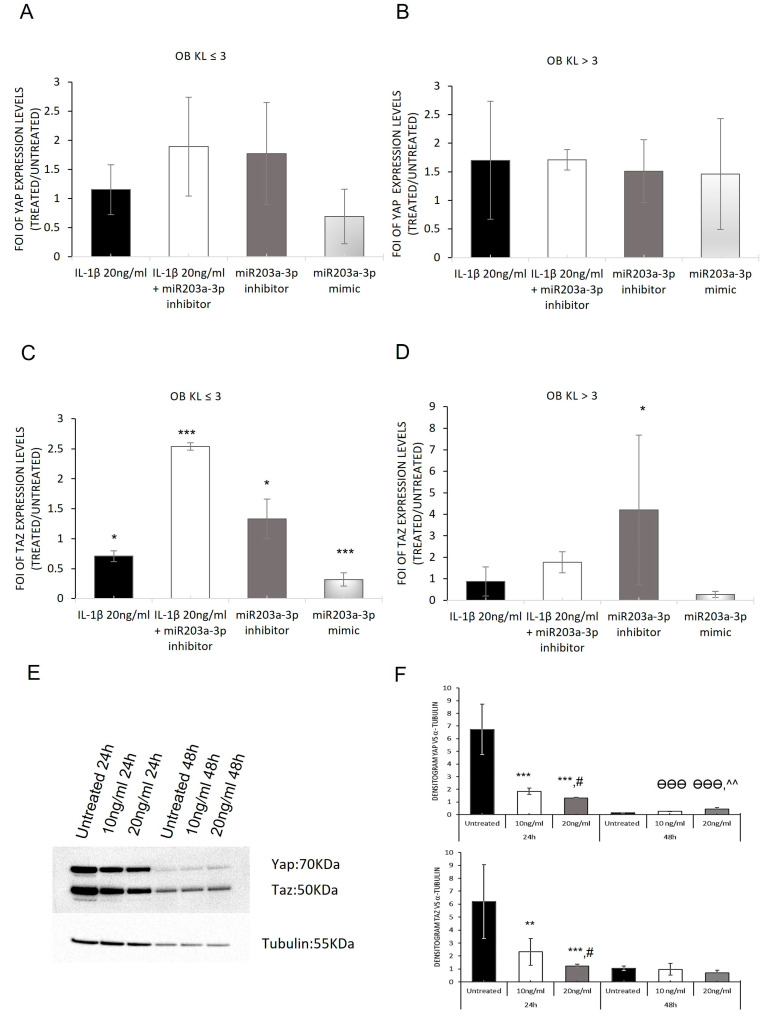
Modulation of YAP and TAZ: from gain and loss of function study to hMSCs treated with IL-1β. qRT-PCR analysis of *YAP/TAZ* mRNA expression OB-OA-derived KL ≤ 3 (**A**,**C**) and KL > 3 (**B**,**D**) groups treated with IL-1β or miR203a-3p inhibitor or miR203a-3p mimic or after co-treatments with IL-1β and miR203a-3p inhibitor. Data are expressed as fold of induction (FOI) in gene expression (2^−ΔΔCt^) occurring in treated vs. scrambled untreated groups (mean ± SD, n = 4). Western blot and densitogram analyses of YAP and TAZ on hMSCs treated with IL-1β (**E**–**F**). One-way ANOVA (YAP, KL ≤ 3: *F* = 2.77, NS; KL > 3: *F* = 0.71, NS; TAZ, KL ≤ 3: *F* = 107.63, *p* < 0.0005; KL > 3: *F* = 7.78, *p* = 0.001); multiple comparisons (one symbol, *p* < 0.05; two symbols, *p* < 0.005; three symbols, *p* < 0.0005 ) versus: *, untreated 24 h; ^θ^ untreated 48 h; ^#^ IL-1β 10 ng/mL 24 h; ^, IL-1β 10 ng/mL 48 h.

Starting with the achieved evidence—a relationship in the expression of miR203a-3p and the axis SP-1/CX-43 and supported by the recent study about the interaction of CX-43 and YAP in OA disease—the expression of YAP and its related interactor TAZ in OBs derived from OA patients was investigated through a gain and loss of function study, hypothesizing an interaction between miR203a-3p-SP1/CX-43 and YAP/TAZ signaling during OA disease. The data showed that the strong regulation mediated by miR203a-3p during OA disease was suitable in TAZ mRNA expression and related proteins, while YAP mRNA did not appear to be modulated by the presence of IL-1β or by transfections with miR203a-3p mimic or inhibitor (Figure 8A,B) [50]. In fact, *TAZ* mRNA was modulated after miR203a-3p overexpression compared with untreated cells, while an increase in its mRNA expression was observed after miR203a-3p inhibitor transfection or after co-treatments (Figure 8C,D).

To understand the difference in the regulation of the YAP/TAZ gene expression between both primary cell groups, we evaluated its expression on hMSCs treated with IL-1β. Our data showed that the protein complex was downregulated after IL-1β treatments as showed in Figure 8E,F.

## 3. Discussion

By considering the complexity of OA disease, the identification of specific biomarkers to monitor its various stages is still an important research focus and goal to reach. The molecular aspects of OA development and progression were investigated with the aim to provide valuable information for the most appropriate treatment, for the evaluation of the response to the treatment, and eventually for the definition of the predictive markers of the OA progression. MiRNAs were identified as suitable markers for various pathological conditions thanks to their simple availability of the source of analysis and for the simple method of their isolation and identification [51,52,53,54,55,56].

In the present study, we investigated the role of miR203a-3p in OA disease as a possible predictive biomarker of inflammatory aggressiveness and OA progression. MiR-203a-3p was mainly identified in various malignant tumors, in which was displayed a controversial role: it was upregulated in some tumors compared with healthy tissues and downregulated in others [13,33,57,58,59,60]. Recently, it was identified in bone as a mediator of cartilage degradation, synovial inflammation response, and OB dedifferentiation, even though evidence in OA progression is still poorly understood [2,22,44,61].

Through the investigation performed in OBs derived from OA patients with different KL grades (KL ≤ 3 and KL > 3) and in hMSCs treated with IL-1β to mimic the in vitro inflammatory conditions of OA, we identified the role of miR230a-3p and its relative targets during the various stages of disease progression. First, a difference in the inflammatory state of cells derived from OA patients was highlighted: OBs derived from patients with KL > 3 showed higher levels of IL-1β, IL6, and IL8 (mRNA and proteins) compared with those derived from patients with KL ≤ 3 (Figure 1). Second, the relationship between inflammatory progression in terms of IL-1β release and miR203a-3p expression was demonstrated. Recent studies suggested a double link between them: IL-1β was able to induce the expression of miRNAs, and in the same manner, miR203a-3p improved the expression of IL-1β probably blocking its transcriptional repressor or via NF-κB signaling [8,33]. Current data showed that miR203a-3p was upregulated in OBs with a low level of OA (KL ≤ 3) compared with a severe level (KL > 3), and the gain and loss of function studies suggested IL-1β was able to induce the expression of miRNAs in both cells (Figure 2) and consequently downregulate its target, TRPV-4 (Figure 3).

Through the gain and loss of function studies (Figure 4), it was identified that the presence of IL-1β alone or in combination with miR203a-3p inhibitor induced differently the upregulation of IL6 and IL8 mRNAs and proteins in OB-KL ≤ 3 and KL > 3 groups compared with untreated cells. Moreover, the treatments with inhibitor and mimic also should increase the expression of ILs in terms of mRNA and proteins compared with untreated cells. These data were supported by many studies [38,41,62] reporting that miR203a-3p induced the expression of IL6 (mRNA and protein), modulating its major transcriptional factor NF-Κb [33], and by bioinformatic software analysis revealing interleukin 6 cytokine family signal transducer (IL6ST) as an miR203a-3p target. 

Working with primary cells can complicate the identification of the actor of a phenomenon, causing comprehension to become difficult. To overcome this aspect, we mimic OA disease in vitro using the model of hMSCs treated with IL-1β, to investigate the role of IL-1β as a modulator of IL6 and IL8 expression and as a promoter of miR203a-3p expression. The hMSC OA model analysis revealed that IL-1β was able to induce the expression of miR203a-3p and consequently downregulate its target TRPV-4 (Figure 5) in a different manner during the experimental time point, while the IL6 gene and protein analysis showed that the transcriptional modulation of mRNA did not correspond to the protein release regulation. The methylation analysis of IL6 and IL8 (Figure S3) promoter genes justified these findings; IL-1β treatments induced a lower methylation of both IL promoters during the experimental time point, inducing an increase in transcription and consequently in its protein release [38,39]. Concerning these data, we can hypothesize that miR203a-3p displayed a role at the transcriptional levels of IL6 and IL8 mRNA, but in the presence of IL-1β stimulation, the regulation of the promoters’ methylation overcame the modification induced by miR203a-3p. In our opinion, this was the reason because we found a different modulation of these ILs between the two groups of OB-OA-derived cells.

Subsequently, the interrelated actions of IL-1β and miR203a-3p were investigated in CX-43 expression. In a previous study, we demonstrated in OBs and chondrocytes derived from OA patients that miR31-5p or miR33a-5p regulated CX-43 expression in a different manner based on the grade of OA and the cytotypes. The proteomic investigation suggested a direct role of Cx-43 in the development of OA, through an enrichment of Cx-43 interactors in OA samples compared with control samples. In addition, recent evidence suggested CX-43 is overexpressed in the middle stage of OA, favoring the maintenance of the chondrocytes in the fibrotic phenotype state leading to cartilage degeneration bringing on joint degeneration [46]. The preliminary current data reported that hMSCs treated with 20 ng/mL of IL-1β showed a significant increase in CX-43 mRNA and protein expression after 48 h of stimulation, while only an upregulation of CX-43 mRNA was found in OBs derived from the KL ≤ 3 group compared with the KL > 3 sample, suggesting the role of miR203a-3p as a regulator of CX-43 expression during the early stage of OA (Figure 6).

To further deepen the comprehension of miR203a-3p’s role, the expression of Sp-1, a transcriptional promoter of the CX-43 gene, was assayed [63]. In OA disease, it was demonstrated that the downregulation of CX-43 in the membrane induced the reduction of Sp-1 recruitment to CxREs (CT-rich connexin response elements) and consequently caused less phosphorylation by the ERK cascade leading, for example, to the alteration of the cell phenotype. Our results highlighted that SP-1 was upregulated and able to modulate its target ALP, suggesting that the hMSCs retained their differentiation ability (Figure 7) and confirming that IL-1β induced the same phenomena activated during OA progression. The gain and loss of function studies revealed that the presence or lack of miR203a-3p altered the expression of SP-1 only in the OBs derived from the KL ≤ 3 group (Figure 8). These data allowed us to hypothesize the involvement of miR203a-3p in the regulation of SP-1 mRNAs during the early stage of OA but not in the severe stage of OA. From observing all data described until now, it seems that a direct link among Sp-1, CX-43, and miR203a-3p exists that should be investigated in planned future studies.

Nevertheless, encouraged by this evidence, we evaluated the possible role of miR203a-3p induced by IL-1β in the regulation of YAP and TAZ expression during OA progression. Recently, the correlation was reported between CX-43 and YAP in astroglial mesenchymal transition, wherein downregulation of Cx-43 improved the CX-43/YAP complex dissociation and nuclear translocation of YAP [47] and consequently the activation of redifferentiation process in the target cells [64]. Through the gain and loss of function study we revealed that TAZ was upregulated after inhibitor transfection or co-treatments with IL-1β in OBs, while the mimic and IL-1β treatments induced the downregulation of its expression. In addition, in both OB groups, no significative variations in YAP expression were identified. On the contrary, the hMSCs treated with IL-1β showed a downregulation of YAP and TAZ, during the experimental times (Figure 8). This controversial expression revealed in OB groups was supported by the new evidence about the novel mechanism of YAP/TAZ regulation; it was demonstrated that YAP inversely regulates the abundance of TAZ protein by proteasomal degradation. Interestingly, this phenomenon was unidirectional since TAZ expression did not affect YAP abundance and TAZ degradation was a consequence of YAP-targeted gene transcription involving TEAD factors [50]. With regard to current YAP/TAZ results, we hypothesized that the different modulation on these proteins could follow this mechanism of regulation during OA progression.

**Figure 9:**
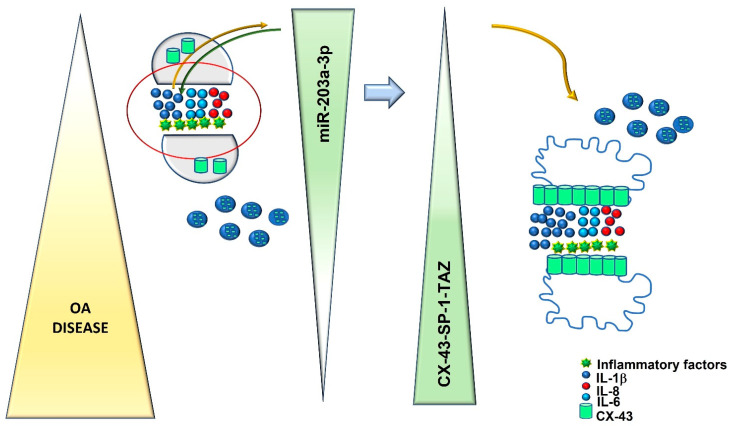
Graphical representation of our hypothesis of the involvement of miR203a-3p in OA disease progression (left part represents the early stage of OA, while the right part shows the middle stage of OA). The yellow and green arrows highlight the co-regulation between IL-1β and miR203a-3p that supports the expression of the proinflammatory factors IL-6 and IL-8 and the trafficking of CX-43 from cells to exosomes, in order to improve the aggressiveness of OA during the time period.

Finally, the current data highlighted the possibility to identify a role of specific molecules as biomarkers in the OA progression (Figure 9). During the early stage of OA, the disease niche was characterized by inflammatory microenvironments in which IL-1β displayed a central role. IL-1β induced the expression of miR203a-3p that improved the expression of IL-1β, self-creating a loop of activation and inducing the expression of IL-6 and IL-8. The progression of inflammatory conditions improved the evolution of OA, favoring probably a different methylation of miR203a-3p promoter or its precursor pre-miR203, leading to the downregulation of its expression during the OA progression. Regarding this hypothesis, many studies suggested the role of miR203a-3p as a tumor suppressor based on the methylation state of its promoter, which encouraged our idea [33,58,59,65,66,67,68,69]. At the middle stage of OA, the downregulation of miR203a-3p and the increase in inflammatory factors improved the expression of CX-43 and SP-1, allowing probably the release of CX-43 by exosomes, as recently demonstrated [19], or the activation of the EMT process or Hyppo signaling, as suggested by the upregulation of TAZ expression after miR203a-3p inhibitor transfection, preparing the cells to go through a fibrotic process that leads to the destruction of the joint, a typical feature of the severe state of OA (Figure 9)

## 4. Material and Methods

### 4.1. Cell Cultures and Reagents

Osteoblasts (OBs) were isolated from waste surgical joint tissues (Protocol ID: CE AVEC 287/2018/Sper/IOR) of patients aged >40 years hospitalized for surgery of i) endoplasty or arthroplasty for OA with Kellgren and Lawrence (KL) grading > 3 (n = 4 patients) or ii) joint fractures (e.g., femoral neck fractures) requiring the implantation of a joint prosthesis that showed KL grading ≤ 3 (n = 4 patients). The demographic and clinical data of selected patients are reported in Table 1. OBs were isolated according to the appropriate protocol and maintained in culture in specific differentiated mediums (Osteoblast Growth Medium, iXCells Biotechnologies MD-0054).

Commercially available human mesenchymal stromal cells (hMSCs; Lonza, Walkersville, MD, USA) were cultured in Mesenchymal Stem Cell Growth Medium (MSCGM™ Bullet Kit, Lonza, Walkersville, MD, USA). The culture medium was changed every three days, and cells were split at 70–80% of confluence using StemProAccutase (Gibco by Life Technologies Italia, Monza, Italy). All cells were maintained in culture in a humidified atmosphere of 5% CO_2_ at 37 °C until the third passages; then, the cells were plated to perform the following assays.

### 4.2. Interleukin 1 Beta (IL-1β) Cell Treatments

For the IL-1β treatment, hMSCs were seeded at 100,000 cells/cm^2^ and treated with 10 or 20 ng/mL of IL-1β (200-01B, Preproteck) for 24 and 48 h to perform assay analyses.

### 4.3. Cell Transfection

For cell transfection, Attractene Transfection Reagent (cat. number 1051531, Qiagen Srl, Milan, Italy) was used following the manufacturer’s indication. Briefly, cells seeded at 150,000 cells/cm^2^ were transfected with 30 pmol/mL of has-mir-203a-3p mimic (MC10152-MIMAT0000264mirVana miRNA mimics Life Technologies Italia), has-miR203a-3p inhibitor (MH10152-MIMAT0000264, mirVana miRNA inhibitors-Life technologies Italia), and scrambled negative controls (4464058mirVana negative control Life Technologies, Monza, Italy) for 24 h. These last controls are negative controls of tested miRNA mimics, and the target gene expressions from the negative control-transfected samples were used as baseline values for the evaluation of the effects of the control and experimental miRNA mimic or inhibitor on target gene expression. At each experimental time the cells were processed for the following assays.

### 4.4. RNA Extraction and Real-Time PCR

Total RNA was extracted using the commercially available NUCLEOZOL (FC140400T NucleoProtect RNA), according to the manufacturer’s instructions. RNA was reverse transcribed to cDNA using the High-Capacity cDNA Reverse Transcription Kit (Applied Biosystems, ThermoFisher Scientific, Waltham, MA, USA). Quantitative RT-PCR (qRT-PCR) analysis was performed in duplicates for each data point, using custom-made primers (Invitrogen, Life Technologies Italia) (Table 2) and Qiagen Primers (Table 3). The mean threshold cycle was used for the calculation of relative expression using the Livak method against *ACTB* [70,71]. For miRNA expression, 250 ng of RNA was reverse transcribed according to the manufacturer’s instructions (cat. number 4366596, TaqMan MicroRNA Reverse Transcription, Applied Biosystems, ThermoFisher Scientific, Waltham, MA, USA). TaqMan probes were used to analyze miR203a-3p (MI0000283-000507 Applied Biosystem, ThermoFisher Scientific, Waltham, MA, USA). Changes in the target miRNA content were calculated in relation to the housekeeping RNU6-1“RNA, U6 small nuclear 1” (4427975 Applied Biosystems, ThermoFisher Scientific, Waltham, MA, USA).

### 4.5. ELISA Assay

Protein release was measured in the culture medium for IL-6 and IL-8 using (SEA079Hu for IL-6 and SEA080Hu for IL-8; Cloud-Clone Corp, 1304 Langham Creek Dr Ste 164, Houston, TX, USA) according to the manufacturer’s instructions. The data were expressed as fold of change (FOI) of protein release relative to the untreated group or in pg/mL amount for each sample tested.

### 4.6. Western Blot Analysis

SDS-PAGE and Western blotting (WB) were performed according to standard protocols. Briefly, after transfection, cells were lysed in lysis buffer containing 15 mM Tris/HCl pH7.5, 120 mM NaCl, 25 mM KCl, 1 mM EDTA, 0.5% Triton X100, and Halt Protease Inhibitor Single-Use cocktail (100X, Fisher Scientific Italia, Rodano, Italy). Whole lysate (15 µg per lane) was separated using 4–12% NovexBis-Tris SDS-acrylamide gels (Invitrogen, Life Technologies Italia), electro-transferred on nitrocellulose membranes (Bio-Rad Laboratories Srl, Segrate, Milan, Italy), and immunoblotted with the appropriate antibodies. Antibodies against the following proteins were used: Sp1 (Sp1 (E-3) Antibody, sc-17824, Santa Cruz Biotechnology, Inc, Dallas, Texas, USA), Cx-43 (connexin 43 (F-7) Antibody, sc-271837, Santa Cruz Biotechnology, Inc, Dallas, Texas, USA), TRPV4 (TRPV4 Antibody #65893, Cell Signaling Technology), and α-Tubulin (monoclonal anti-α-Tubulin (TU-02), sc8035, Santa Cruz Biotechnology, Inc. Dallas, Texas, USA). All secondary antibodies were obtained from Fisher Scientific Italia. Immunofluorescence was detected and analyzed using a CCD high-resolution and high-sensitivity detection technology (ChemiDoc™ XRS+ System, Bio-Rad Laboratories Srl).

### 4.7. MSRE PCR Analysis

The isolation of the genomic DNA of MSCs, under different treatments (IL-1β at 10 ng/mL or 20 ng/mL), was carried out with the PureLink Genomic DNA mini-Kit (Invitrogen™, Waltham, MA, USA). The DNA was quantified using the Nanodrop 2000 spectrophotometer (ThermoFisher Scientific, Waltham, MA, USA), and the integrity was analyzed using electrophoresis on 0.8% agarose gel. The methylation sensitive restriction endonuclease–PCR (MSRE–PCR) analysis was performed to determine the methylation status of the CpG-rich sites, present in the proximal promotor regions of IL-6 and IL-8. The experiments were carried out as described elsewhere [38,72,73]. In brief, the PCR products were analyzed by 2% agarose gel electrophoresis, visualized by Gel Red staining (Biotium, Hayward, CA, USA) in a ChemiDoc apparatus (Bio-Rad Laboratories, Hercules, CA, USA), and densitometric analyses were obtained using the “Image Lab” application (version 5.2.1) of Bio-Rad Laboratories (Hercules, CA, USA).

### 4.8. Statistical Analysis

The statistical analysis was performed by using R software v.4.2.1 [71]. One- or two-way ANOVA was used to evaluate the significant effects and/or interactions of selected factors (“treatment” for one- and two-way and “experimental time” for two-way) on normally distributed (Shapiro–Wilk test) data with homogeneity of variance (Levene test). Then, selected pairwise multiple comparisons with *p*-values adjusted according to the Sidak–Holm or Dunnett method were carried out.

## 5. Conclusions

The current results suggest that the increased expression of miR203a-3p, induced by the presence of pro-inflammatory mediators, has in turn an active role in inflammation, cell dedifferentiation, and transition, rendering cells unable to control the expression of SP-1, CX-43, and TAZ. These phenomena have led to the reduction of miR203a-3p expression, which, probably, will progressively degrade in a manner directly proportional to the increase in the severity of the disease, leading to the development of a microenvironment altered by a chronic inflammatory process and by an aberrant cellular dedifferentiation, until the final joint degeneration. To confirm this hypothesis, other investigations will be performed on the blood of OA patients from which will be isolated circulating miRNAs and exosomes, to evaluate their miR203a-3p enrichment and the proinflammatory cytokine amount. In addition, to validate our idea about the correlation between the inflammatory aggressiveness and miR203a-3p expression, we will perform the lymphocyte immunophenotyping by FACS analysis, to identify the possible enrichment of a specific lymphocyte subpopulation during the various steps of OA progression.

These studies will support the protective role of miR203a-3p and its possible use as a predictive biomarker in OA progression.

## Figures and Tables

**Table 1:** Demographic and clinical data of selected patients.

	OA KL ≤ 3 (n = 4)	OA KL > 3 (n = 4)
Age (yrs)	60 ± 6	64 ± 11
Weight (kg)	66.8 ± 9.7	80.5 ± 24.3
BMI (kg/m²)	24.3 ± 2.8	27.1 ± 4.5
Other pathologies	COPD Hashimoto thyroiditisBreast cancer	Hypertension Gastroesophageal relux
WBC (x10³/μL)	5.89 ± 0.67	7.62 ± 1.01
CRP (mg/L)	0.20 ± 0.07	0.28 ± 0.11

**Table 2:** List of gene primers used to study gene expression profiling. Their expression was normalized to the b-actin housekeeping gene.

*Gene*	Primer Forward	Primer Reverse
*SP-1* *“Specific protein 1”*	GCCTCCAGACCATTAACCTCAGT	GCTCCATGATCACCTGGGGCAT
*CX-43* *“Connexin43”*	GAACTCAAGGTTGCCCAAAC	TTAGAGATGGTGCTTCCCG
***Reference Gene***		
*ACTB* *“Beta-Actin”*	ATCAAGATCATTGCTCCTCCTGA	CTGCTTGCTGATCCACATCTG

**Table 3:** List of gene Qiagen primers used to study gene expression profiling. Their expression was normalized to the b-actin reference gene.

*Gene*	Qiagen Number
*IL-1β* Hs_IL-1β_1_SG QuantiTect Primer Assay	QT00021385
*IL-6* Hs_IL-6_1_SG QuantiTect Primer Assay	QT00083720
*IL-8* Hs IL-8_1_SG QuantiTect Primer Assay	QT00083756
*ALPL* Hs_ALPL_1_SG QuantiTect Primer Assay	QT00012957
*TRPV4* Hs_TRPV4_1_SG QuantiTect Primer Assay	QT00077217
***Reference Gene***	
*ACTB* Hs_ACTB_1_SG QuantiTect Primer Assay	QT00095431

## Data Availability

The data presented in this study are available on request from the corresponding author.

## Supplementary Materials

The following are available online at https://www.mdpi.com/article/10.3390/ijms24054316/s1.
