# Supplementary material for: Timing Expression of miR203a-3p during OA Disease: Preliminary In Vitro Evidence
Source: Int J Mol Sci. 2023 Feb 21;24(5):4316. doi: 10.3390/ijms24054316 (PMC10002134; doi:10.3390/ijms24054316)
Supplement: Supplementary file 1 [file ijms-24-04316-s001.zip › ijms-2089691-supplementary.pdf]

Supplementary

S1

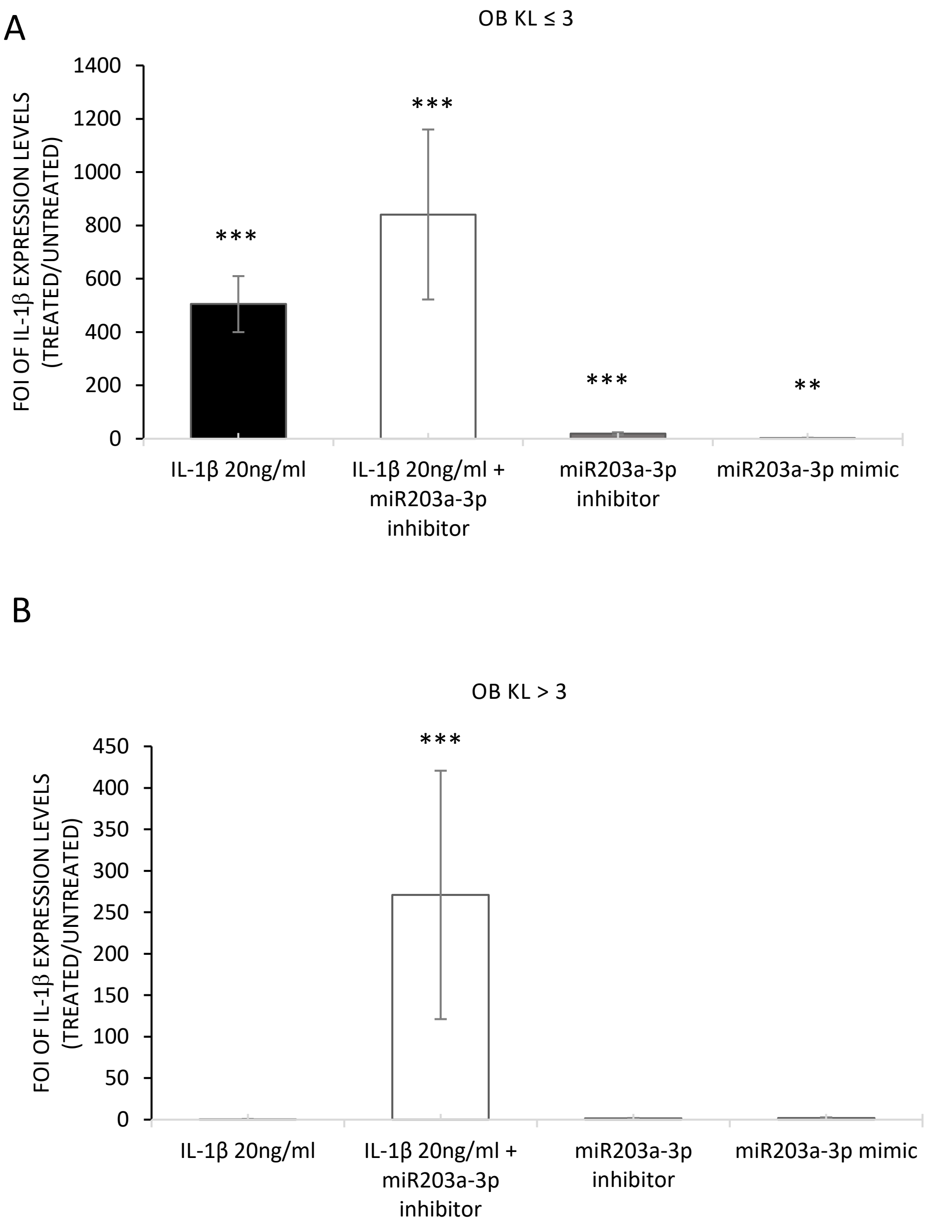

Figure S1. qRT-PCR analysis of IL-1β expression on OB derived by KL>3 group (A) and KL ≤3 group (B) treated :with IL-1β , after transfection with miR203a-3p inhibitor, after co-treatments IL-1β and miR203a-3p inhibitor. Data are reported as FOI of expression between treated and untreated samples. (Mean ± SD, n=4). Student’s t- test: 1 symbol, $p < 0.05$; 2 symbols, $p < 0.005$; 3 symbols, $p < 0.0005$.

Supplementary

S2

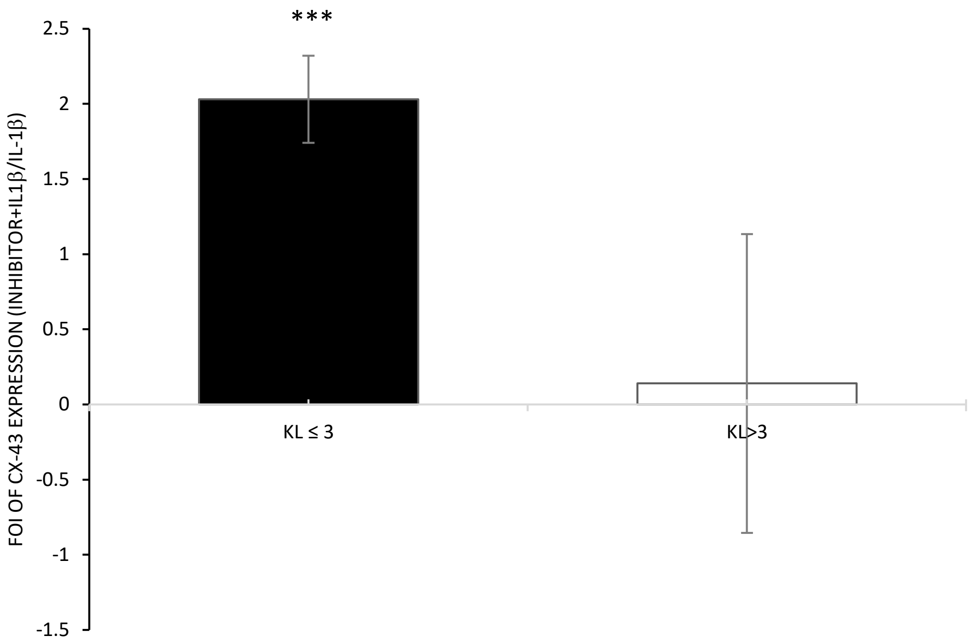

Figure S2. qRT-PCR analysis of CX-43 expression on OB derived by KL>3 group and KL ≤3 group treated :with IL-1β , after transfection with miR203a-3p inhibitor, after co-treatments IL-1β and miR203a-3p inhibitor. Data are reported as FOI of expression between interleukin treated and inhibitor miR203a-3p transfected cells versus interleukin treated cells (Mean ± SD, n=4). Student's t- test: 1 symbol, $p < 0.05$; 2 symbols, $p < 0.005$; 3 symbols, $p < 0.0005$.

S3

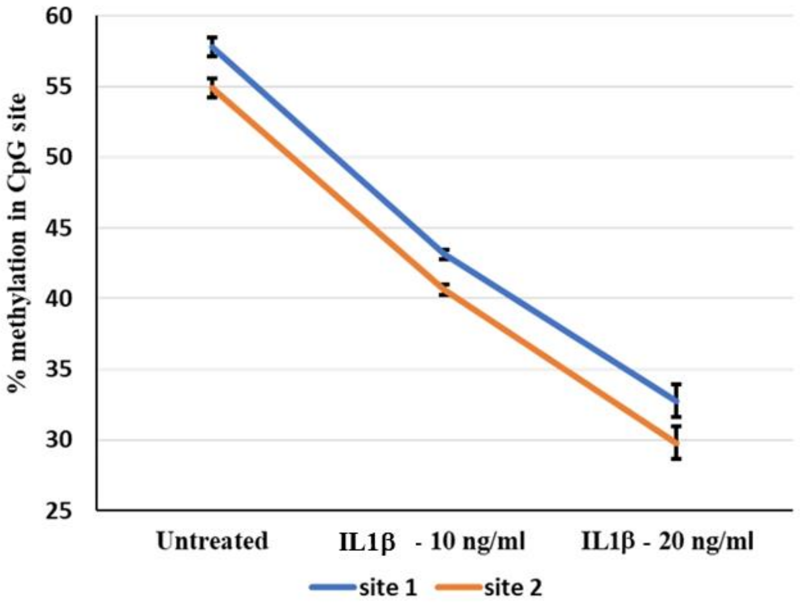

Figure S3: Percentage of methylation of two methyl-sensible restriction sites through MSRE-PCR analyses of IL-8 promoter of MSCs after 48 hrs under to the following treatments: Untreated; IL-1β (10 ng/ml); IL-1b (20 ng/ml); (Mean ± SD, n = 3, duplicates).
